# Loratadine bioavailability via buccal transferosomal gel: formulation, statistical optimization, *in vitro*/*in vivo* characterization, and pharmacokinetics in human volunteers

**DOI:** 10.1080/10717544.2017.1321061

**Published:** 2017-05-08

**Authors:** Mohammed H. Elkomy, Shahira F. El Menshawe, Heba A. Abou-Taleb, Marwa H. Elkarmalawy

**Affiliations:** 1 Department of Pharmaceutics and Industrial Pharmacy, Beni-Suef University, Beni-Suef, Egypt and; 2 Department of Pharmaceutics and Clinical Pharmacy, Nahda University, Beni-Suef, Egypt

**Keywords:** Loratadine, transferosomes, buccal, mucoadhesive gel, bioavailability

## Abstract

Loratadine (LTD) is an antihistaminic drug that suffers limited solubility, poor oral bioavailability (owing to extensive first-pass metabolism), and highly variable oral absorption. This study was undertaken to develop and statistically optimize transfersomal gel for transbuccal delivery of LTD. Transfersomes bearing LTD were prepared by conventional thin film hydration method and optimized using sequential Quality-by-Design approach that involved Placket–Burman design for screening followed by constrained simplex-centroid design for optimization of a Tween-80/Span-60/Span-80 mixture. The transferosomes were characterized for entrapment efficiency, particle size, and shape. Optimized transferosomes were incorporated in a mucoadhesive gel. The gel was characterized for rheology, *ex vivo* permeation across chicken pouch buccal mucosa, *in vitro* release, and mucoadhesion. Pharmacokinetic behavior of LTD formulations was investigated in healthy volunteers following administration of a single 10-mg dose. Optimal transferosomes characterized by submicron size (380 nm), spherical shape and adequate loading capacity (60%) were obtained by using quasi-equal ratio surfactant mixture. In terms of amount permeated, percentage released, and mucoadhesion time, the transferosomal gel proved superior to control, transferosome-free gel. Bioavailability of the transferosomal gel was comparable to Claritin® oral tablets. However, inter-individual variability in *C*_max_ and AUC was reduced by 76 and 90%, respectively, when the buccal gel was used. Linear Correlation of *in vitro* release with *in vivo* buccal absorption fractions was established with excellent correlation coefficient (*R^2^*>0.97). In summary, a novel buccal delivery system for LTD was developed. However, further clinical investigation is warranted to evaluate its therapeutic effectiveness and utility.

## Introduction

Loratadine (LTD) is a non-sedative second-generation antihistaminic drug that is selective for peripheral H1-receptor in the nose and conjunctivae, thus relieving itching, congestion, rhinorrhea, tearing, and sneezing symptoms. LTD belongs to class II of the Biopharmaceutics Classification System (BCS). LTD is rapidly absorbed following oral administration. However, LTD oral absorption is limited and highly variable. Owing to extensive first-pass metabolism, LTD oral bioavailability does not exceed 40% (Arya et al., 2013). LTD is a weakly ionizable base with pH-dependent solubility; solubility decreases exponentially with pH increase (El-Hammadi & Awad, 2012). Hence, LTD oral absorption shows high degree of intra- and inter-subject variability and lack of dose proportionality (Patil & Paradkar, 2006).

Attempts to mitigate LTD poor oral bioavailability have relied on two strategies; (i) improving drug’s limited solubility in gastrointestinal fluid through β-cyclodextrin complexation (Szabados-Nacsa et al., 2011) and self-microemulsifying drug delivery systems (Li et al., 2015), and (ii) bypassing hepatic first-pass metabolism through non-oral administration (Cho et al., 2009; Song & Shin, 2009; Singh et al., 2013; Chakraborty et al., 2014; Kumria et al., 2014). Published reports (Chakraborty et al., 2014) suggested the buccal administration as an alternative route for systemic delivery of LTD. It seems that LTD could benefit from the high permeability of the oral mucosa and low degradation risk. Moreover, patient compliance with buccal administration is almost guaranteed because of convenient self-medication and easy dosage form administration and removal. Besides, the buccal mucosa is characterized by high vasculature and the epithelium of the oral cavity has shorter recovery time as compared to other mucosal epithelial surfaces (Kumria et al., 2014).

Mucoadhesive buccal wafers significantly improved LTD bioavailability in a rabbit animal model (Chakraborty et al., 2014). Motivated by the promising findings in literature (Chakraborty et al., 2014), we decided to exploit the buccal administration as an alternative route to LTD ingestion. However, we have elaborated on the work of Chakraborty et al. (2014) by investigating potentials of transferosomes as carriers for LTD transbuccal delivery. Transfersomes are the first generation of ultra-deformable vesicles that are mainly composed of a combination of phospholipids and an edge activator which is often a single-chain surfactant that destabilizes the lipid bilayers of the vesicles and increases the deformability of the bilayers (Cevc, 1996). The high deformability and hydration driving force of the ultra-deformable vesicles prompt drug-loaded vesicles to move across the skin barrier to reach deeper dermal tissues and even the systemic circulation, and minimize the possibility of their rupture especially when applied onto the skin (Cevc, 1996).

This study was undertaken to develop and statistically optimize transfersomal gel for transbuccal delivery of LTD. A sequential, computer aided Quality-by-Design approach was adopted to address the aforementioned objective. Initially, possible formulation and processing variables were screened via Placket–Burman to ensure their relevance to the aim. In a subsequent stage, recognized significant variables were correlated to selected response variables via polynomial equations. In a later stage, equations generated response surfaces together with desirability index were mingled to identify an optimum transferosomal formulation. The optimized formulation was incorporated into a gel-based vehicle to facilitate buccal application. The constructed drug delivery system was exposed to *in vitro*/*in vivo* performance evaluation tests including bioavailability studies in human volunteers.

## Materials and methods

### Materials

LTD was purchased from Sigma-Aldrich (Cairo, Egypt). Phosphatidyle choline, sodium cholate, sodium deoxy cholate, ploxamar-188 and isopropyl myristate were purchased from Acros Organics (Cairo, Egypt). Span-80 and Span-60 were purchased from Oxford Laboratory Reagent (Cairo, Egypt). Dialysis bags with molecular weight cut off of 12 000 Da were purchased from Sigma-Aldrich. Carbopol-940, Tween-80 and Tween-20 were purchased from El-Nasr Pharmaceutical Chemical Company (Cairo, Egypt). All other reagents were of analytical grade and were commercially available.

### Preparation of LTD-loaded transfersomes

LTD-loaded transfersomal (LTD-TRS) vesicles were prepared by a thin film hydration method (Ahad et al., 2016). Phosphatidylcholine, edge activator, and the drug were dissolved in a clean, dry, round bottom flask, containing chloroform. Solvent was removed by rotary evaporation (Heidolph Laborota 4000 Series, Heizbad, Germany) under pressure at temperature 60 °C. The formed lipid film was then hydrated with a 10 ml solution of phosphate buffer and hydrophilic surfactant under rotation at 60 rpm for 10 min at room temperature. Then, the flask containing the hydrated film was sonicated using UH-100B ultrasonic processor sonicator (Tianjin Automatic Science Instrument Ltd, Nanyang, China) to convert large multilamellar vesicles into smaller vesicles.

### Preliminary screening studies

The method for preparation of LTD-loaded transferosomes involved several variables. To screen critical variables that affect entrapment efficiency and particle size of loaded transferosome formulations a six-factor 12-run Plackett–Burman Design was implemented. The variables studied were: type of hydrophobic surfactant (Span-60 and Span-80), ratio of lipid and edge activator to surfactant (2:1 and 4:1), sonication time (10 and 30 min), type of edge activator (sodium cholate and sodium deoxy cholate), ratio of lipid to edge activator (2:1 and 5:1), and type of hydrophilic surfactant (Tween-20 and Tween-80). Composition of LTD-TRS formulations according to the design is shown in Supplementary Table S1. Data collected from the design were analyzed using analysis of variance. The Plackett–Burman design allows estimation of a main effect for each independent variable. To increase the error degrees of freedom, the design was replicated twice.

### Quantification of LTD by HPLC analysis

Quantitative analyses of LTD were achieved by high-performance liquid chromatography (HPLC) method using quaternary gradient HPLC (Knauer, Berlin, Germany) with Smartline Manager 5000 Degasser and Smartline Pump 1000. The method was adopted from a published report (Kunicki, 2001) with modification. LTD was chromatographed on Thermo Scientific ODS C18 RP column (1504.6 × 5 mm) in isocratic mode, at 30° C. The optimized degassed mobile phase was a 35:45:20 (v/v) mixture of acetonitrile, methanol and a phosphate buffer solution (0.01 M, pH 7.2 ± 0.1, adjusted with dilute orthophosphoric acid). The flow rate was 1.0 mL/min with injection volume of 20 mL and the analyte was monitored at 254 nm (UV detector). The reliability of the analysis method was determined by obtaining linearity and accuracy.

### Drug content and entrapment efficiency determination

Drug content and entrapment efficiency (EE) was estimated by separating free LTD from LTD-TRS using cooling centrifuge method (Ahmed, 2015). Briefly, the transfersomal formulation was centrifuged at 14 000 rpm for 60 min at 4 °C (Sigma Laborzentrifugen D-37520, Osterode-am-Harz, Germany). The precipitated transferosomes were washed with PBS at pH 7.4 twice then, the clear fraction (supernatant) was separated each time from transferosomes and filtered with a 0.45 μm nylon syringe filter then assayed for free non-entrapped drug using the HPLC method described above. The %EE of LTD was calculated using Equation (1):

(1) EE %=Total drug concentration-free drug concentrationTotal drug concentration×100

### Determination of particle size

The dynamic light scattering (DLS) was used to determine the mean particle size (PS) of LTD-TRS. Briefly, the samples were diluted with deionized water, adjusted to 25 °C then subjected to laser light with an incident laser beam of 633 nm at a scattering angle of 90° using the Malvern Zetasizer Nano 6.01 (Malvern Instruments GmbH, Herrenberg, Germany) (Abdellatif & Tawfeek, 2015). At least three independent samples were taken, and the PS was measured at least three times.

### Optimization studies

#### Constrained simplex-centroid design

**Figure 1.**
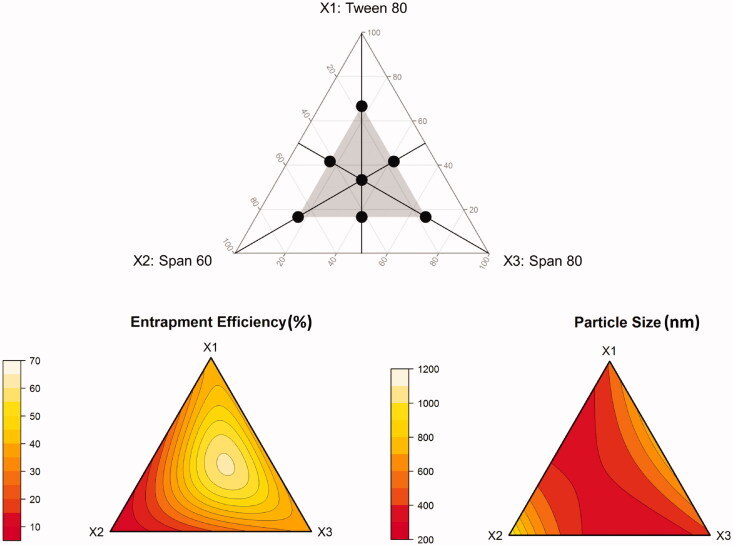
Constrained simplex-centroid design for optimization of a ternary surfactant mixture (upper panel), and ternary response surface plots showing the effect of mixture composition on measured responses (lower panel).

A mixture design was adopted to optimize a ternary surfactant mixture composed of Tween-80 (*X*_1_), Span-60 (*X*_2_), and Span-80 (*X*_3_). In these designs, the total amount of surfactant mixture was kept constant, while changing the amount of each of the surfactants simultaneously. A standard simplex-centroid design for a three-component system is an equilateral triangle in two-dimensions. The design involves three corner points, one at each vertex: (1,0,0), (0,1,0), (0,0,1), three midpoints, one at the halfway to each vertex: (1/2,1/2,0), (1/2,0,1/2), (0,1/2,1/2), and one center point (1/3,1/3,1/3). In this study, the simplex-centroid design was constrained so that all components are represented in all runs. To do so, the corner and midpoints, where at least one component was missing, were shifted to half of the corresponding distance to the design center point. The shifted corner points are: (1/6,1/6,2/3), (1/6,2/3,1/6), (2/3,1/6,1/6). The shifted midpoints are: (5/12,5/12,1/6), (5/12,1/6,5/12), (1/6,5/12,5/12). The constrained simplex-centroid design used for optimization of the surfactant mixture is shown in Figure 1 (upper panel). The composition of the formulations associated with the proposed mixtures is depicted in Supplementary Table S2. All experiments were performed in two replicates. The center point was replicated twice.

#### Multiple linear regression

Three regression models were fitted to the data collected for each response from the eight mixture runs. The models were (Scheffé, 1958):

First-order (linear):

(2) $$f\left(Y\right)=b_{1}X_{1}+b_{2}X_{2}+b_{3}X_{3}$$

Second-order (quadratic):

(3) $$f\left(Y\right)=b_{1}X_{1}+b_{2}X_{2}+b_{3}X_{3}+b_{12}X_{1}X_{2}+b_{13}X_{1}X_{3}+b_{23}X_{2}X_{3}$$

Special third-order (special cubic):

(4) $$f\left(Y\right)=b_{1}X_{1}+b_{2}X_{2}+b_{3}X_{3}+b_{12}X_{1}X_{2}+b_{13}X_{1}X_{3}\;+b_{23}X_{2}X_{3}+b_{123}X_{1}X_{2}X_{3}$$

where, *Y* is the measured response associated with each mixture combination, *f*(.) is a functional form of the response, *X*_1_ to *X*_3_ are the coded mixture components, and b_1_ to b_123_ are the regression coefficients.

The coefficients b_1_–b_3_ represent the expected response to a pure blend of the corresponding component, b_12_–b_23_ the effect of blending between each two mixture components, and b_123_ the effect of blending between the three mixture components. A positive sign on b_1_–b_3_ indicates bigger response on increasing the component level, whereas a negative sign indicates smaller response on increasing the component level. If positive, the coefficients b_12_–b_23_, and b_123_ indicate that the components enhance the effect of each other (synergism). If negative, the components reduce the effect of each other (antagonism). If insignificant, the effect of component blending is purely additive. In terms of response surface, b_1_–b_3_ represent the expected height of the surface at the vertex of each of the components, b_12_–b_23_ the expected amount of quadratic curvature along the edge of the simplex region consisting of binary mixtures, and b_123_ the degree of flexibility that a quadratic surface needs to accommodate the observed responses around the center of the simplex region consisting of the ternary mixture.

Adequacy of the final models was checked using analysis of variance (ANOVA), adjusted multiple correlation coefficient (adjusted *R^2^*), goodness-of-fit plots, residual plots, and Q-Qnorm plots. Conclusions were drawn from the magnitude and sign of the coefficients of the fitted polynomial equations. Two-dimensional simplex surface plots were created and visually evaluated.

#### Desirability function

To identify the optimal composition of the ternary surfactant mixture to be incorporated in transferosome preparations, a desirability index approach (Aboud et al., 2016; Elkomy et al., 2017) based on the work of Derringer and Suich (1980) was used. The computed desirability index compiles all responses into a single variable that is optimized by searching through a grid of mixture plausible combinations under the constraint that mixture components fractions add up to one. Preset criteria for the search process were: (i) maximize EE within the range 50–70% and (ii) minimize PS within the range 350–800 nm.

#### Statistical analysis and software

ANOVA, multiple linear regression (MLR), as well as desirability function maximization analyzes were performed in R version 3.10 (R Core Team 2014, Vienna, Austria). Plackett–Burman design, constrained simplex-centroid design, design plot, diagnostic plots, and response surface plots were created in R version 3.10 (R Core Team 2014, Vienna, Austria).

### Morphology of LTD-TRS

The optimized LTD-TRS formulation was selected for examining morphological shape of the transfersomes. Before determination, the sample was diluted using distilled water. The size and morphology of the selected formula was examined by transmission electron microscope (TEM) (PHILIPS CM10, Eindhoven, The Netherlands), with an accelerating voltage of 100 kVA. Briefly, a drop of the sample was placed on a carbon coated copper grid and allowed to stand at room temperature for 90 s to form a thin film. Excess of the solution was drained off with a filter paper. Samples were viewed and photomicrographs were taken at suitable magnification (Manconi et al., 2003).

### Preparation and characterization of LTD transferosomal buccal gel (LTD-TRS-gel)

#### Preparation and rheology of LTD-TRS-gel

The optimized LTD-TRS formulation was incorporated into a mucoadhesive gel for buccal administration. The gel base was prepared using carbopol 940 (1% w/v) and ploxamar 188 (2% w/v) as muccoadhesive and gel forming polymers, isopropyl myristate (3% v/v) as penetration enhancer, glycerin (10% v/v) and PEG-400 (5%v/v) as humactants and propyl parabens (0.05% w/v) as preservative. The gel base was prepared using cold method. Control gel containing 10 mg LTD was prepared by dissolving the drug in minimum amounts of ethanol followed by gradual addition to the preformed gel with continuous stirring for 1 h. Similarly, LTD-TRS suspension (equivalent to 10 mg LTD) was incorporated into the gel base. It was stirred for 1 h, and pH was adjusted to 6.8 for both the control and LTD-TRS gels using triethanolamine to obtain gels with adequate consistency suitable for buccal application (Hazzah et al., 2016).

The viscosity of the produced transfersomal and control gels were measured at 25 °C using Brookfield viscometer (RV-TD; Brookfield, MA). The PH value of the produced gels was measured using a digital pH meter (3500 pH meter, Jenway, Staffordshire, UK). The homogeneity of the gel formulations were examined by visual appearance. Small quantity of each gel formulation is placed between the thumb and the index finger, where the consistency of the gel was observed whether homogeneous or not (Abdellatif & Tawfeek, 2015).

#### Muccoadhesion of LTD-TRS-gel

The mucoadhesive performance of LTD-TRS-gel and control gel were measured by determining the force required to detach the gel from chicken pouch membrane using a modified balance method (El Azim et al., 2015).The device used was fabricated in our laboratories. It is composed of a two-sided balance. The left side was composed of a wire suspended vertically and attached to it a small glass with the mucosal membrane fixed on the down side. Below it, there was a movable stage with another mucosal membrane fixed on the top side. The dosage form was applied between two sided mucosa. The right side contained a pan where the weights are added gradually. The force required to detach the two sided mucosa was recorded. The force (dyne/cm^2^) needed to break the adhesive bond per unit area was estimated using the following equation (Hazzah et al., 2016):

(5) F=W×gA

where, *F* is the bioadhesive force in dyne/cm^2^, W is the weight added on the right pan in gram, *g* is the acceleration due to gravity in 980 cm/s^2^ and A is the surface area of the gel layer in cm^2^.

#### In vitro release studies

The *in vitro* release of LTD from transferosomal based buccal gel and control gel were conducted using dialysis method (Abdel-Mottaleb & Lamprecht, 2011). An open ended tube was fixed in USP dissolution apparatus (paddle type) and was sealed from one end with dialysis membrane. Gel equivalent to 10 mg LTD was added into the dialysis tube which was allowed to be inserted into dissolution medium of 900 ml of phosphate buffer at pH 6.8 maintained at temperature of 37 ± 0.5 °C and stirred at 50 rpm. Samples (5 ml) were withdrawn at preplanned time intervals for 24 h and were replaced with equal volume of fresh buffer to maintain sink condition. The amount of drug released was obtained by the HPLC method described above.

In order to determine the release mechanism of LTD from LTD-TRS-gel and control gel, the data acquired from the release studies were fitted to zero-order, first-order, and Higuchi diffusion models. The model was chosen depending on *R^2^* (correlation coefficient) values. The model with the highest value of *R^2^* was considered the best model describing release kinetics.

#### Ex-vivo permeation studies

Permeation studies were performed on LTD-TRS-gel and control gel using the dialysis method described above under the same experimental conditions except for the use of fresh dissected chicken buccal mucosa instead of dialysis membrane. After obtaining the buccal mucosal tissue from a local slaughter house, it was cleaned, washed, and stored at −20 °C. Buccal mucosa was then hydrated with simulated saliva solution and allowed to reach room temperature (25 °C) before proceeding with the permeation experiment. Percentage cumulative amount of LTD permeated through dissected chicken buccal mucosa per unit surface area were plotted against time and analyzed using the following Fick’s equation:

(6) $$J_{\mathrm{ss}}\;=\;\mathrm{dQ}/dtA$$

where, *J*_ss_ is the steady-state flux; *dQ*/*dt* is the permeation rate; where *Q_t_* is the cumulative amount of LTD in the receptor phase at time *t*, and A is the area of application (2 cm^2^).

The enhancement ratio (Er) attributed to transferosome encapsulation was calculated by the following equation:

(7) $$\mathrm{Er}=J_{\mathrm{SS}}\;\mathrm{of}\;\mathrm{transferosmal}\;\mathrm{gel}J_{\mathrm{ss}}\;\mathrm{of}\;\mathrm{control}\;\mathrm{gel}$$

### *In vivo* pharmacokinetic study in healthy human volunteers

#### Study design and subjects

The study was carried out to compare the pharmacokinetics of LTD from the optimized buccal gel containing transferosomal LTD to the marketed tablet Claritin® following administration of a single 10 mg dose of each in a two-treatment, two-period crossover design with 1 week washout period before administration of the other formulation.

The comparative study was carried out on three healthy male volunteers. Their age ranged from 25 to 35 years, with mean body weight of 85 ± 6.7 kg (range: 77–93 kg) and mean height of 182.5 ± 10 cm (range: 177–190 cm). Biochemical examination of the volunteers revealed normal kidney and liver function. The plan and purpose of the study were explained in detail to them. The volunteers were asked to withhold taking medicines one week before the experiment. All subjects fasted for at least 10 h before the study day. The volunteers were asked to sign a written consent and the study was approved by the Beni-Suef University Ethics Committee.

#### Drug administration

The study was performed on two periods: period I, the three volunteers received the conventional commercial tablet Claritine® which is considered a reference standard; period II, volunteers received the LTD-TRS-gel. Clinical protocols for buccal administration of semisolid dosage forms were not available in literature. Therefore, a protocol for buccal administration of midazolam solution in pediatric population (McIntyre et al., 2005) was adopted with modification. The gel formulation was applied to the area between the gum and right sided check. The volunteers were instructed to avoid saliva swallowing for 1 min. Food and drinks (other than water, which was allowed after 2 h) were not allowed until 8 h after dose administration and then a standard lunch and dinner were served to all volunteers according to a time schedule. The presence of gel remainders in the buccal cavity of all volunteers was confirmed by the physician after 120 min of the administration. A wash-out period of one week separated the two periods.

#### Sample collection

A physician supervised the study and was also responsible for the volunteers’ safety and collection of samples. Venous blood samples (5 ml) were collected into heparinized tubes at the following set points: 1, 2, 3, 5, and 24 h after administration of each treatment. Plasma was separated by centrifugation at 3500 rpm for 10 min at 4 °C. The plasma was pipetted directly into 5 ml plastic tubes and stored frozen at −20 °C ready for drug analysis.

#### Chromatographic conditions

Plasma concentrations of LTD were analyzed using a liquid chromatography-tandem mass spectroscopy (LC-MS/MS) method validated according to published guidelines (US FDA 2001). LC system (Agilent 1200, Deutschland GmbH, Waldron, Germany) coupled with Triple Quad mass spectrometer (Agilent Technologies 6420, Deutschland GmbH, Waldron, Germany) was used. The chromatographic separation was carried out on a C18 reverse phase column Inertsil ODS-3 (4.6 mm × 50 cm, dp 5 μm; Sigma-Aldrich). The mobile phase was composed of 20 mM formic acid:methanol:acetonitrile (25:35:40) (v/v/v). The flow rate was set as 0.7 ml/min. Data acquisition was performed working in multiple reactions monitoring mode (MRM) using MassHaunter Software (6400 series, Quadrupole B.07.00). MS parameters for the analysis were as following: ESI source used in the positive ionization mode, capillary voltage: 4 kV; dwell time 200 (ms), fragmentation voltage 135 V, accelerated voltage 7 V and collision energy for LTD and internal standard were 25 and 20 V, respectively. The protonated precursor molecular ions [MH]^+^ of LTD (*m*/*z* = 383) and the internal standard (*m*/*z* = 416) were selected and fragmented. The product ions were, *m*/*z* = 337 for LTD and *m*/*z* = 308 for the internal standard. Source parameters: nitrogen gas as the source, gas temperature 350 °C, gas flow 9 L/min, nebulizer 50 psi and capillary voltage 4 kV.

#### Sample preparation for analysis

Fifty microliters of Rupatadine, as internal standard (from a stock solution of concentration 1000 ng/ml) and 500 μl ammonium hydroxide solution (1 ml 33%NH_3_/25 ml distilled water) was added to each sample (450 μl plasma), vortexed for 1 min and then added to 3 ml ethyl acetate, vortexed again for 1 min then centrifuged for 10 min at 2500 rpm and −4 °C (cooling centrifuge, Sigma, 2-16PK). The upper organic layer was transferred to another tube, filtered through 0.22 mm Millipore filter, then test tube was placed in a concentrator for 45 min at 60 °C till complete evaporation of the solvent. Dry residues were reconstituted with 200 μl mobile phase [20 mM formic acid:methanol:acetonitrile (25:35:40) (v/v/v)], vortexed for 5 min then centrifuged for 10 min at 3000 rpm and finally 10 μl of clear supernatant was injected on the column for analysis.

#### Pharmacokinetic and statistical analysis

Pharmacokinetic parameters were estimated by non-compartmental pharmacokinetic analysis using WinNonlin®. The individual plasma concentration–time curve was used to calculate the maximum drug concentration (*C*_max_) and the time to reach *C*_max_ (*T*_max_), area under the curve from 0 to 24 h (AUC_0–24_) and to infinity (AUC_0–∞_), terminal half-life (*t*_0.5_), and mean residence time (MRT). Results are expressed as mean and standard deviation (SD) values of three volunteers. The %coefficient of variation was calculated as the ratio of the SD to the mean.

The calculated pharmacokinetic parameters of the two treatments were statistically analyzed with ANOVA test for the untransformed data using the software SPSS 17.0 (SPSS Inc., Chicago, IL).

#### In vitro/in vivo correlation

Level A *in vitro*/*in vivo* correlation (IVIVC) was conducted between *in vitro* dissolution and *in vivo* absorption data of LTD-TRS-gel. The fraction of LTD absorbed (F_abs_) following buccal administration of the selected formulation at various times was calculated from the mean plasma concentration–time data of the drug using the equation of Wagner–Nelson (Wagner & Nelson, 1963):

(8) $$F_{abs}=\frac{A_{t}}{A_{\infty }}=\frac{C_{p}+k_{el}\cdot \int_{0}^{t}C_{p}\;dt}{k_{el}\cdot \int_{0}^{\infty }C_{p}\;dt}$$

where, *A_t_* and *A*_∞_ are the amounts of drug absorbed up to time *t* and infinity, respectively; *C_p_* is plasma concentrations; and *k_el_* is the elimination rate constant. The integrals in Equation (8) are the areas under the plasma level–time curve up to time *t* and infinity, respectively.

To calculate the integrals and constant in Equation (1), a system analysis approach was used (Veng-Pedersen, 1988). This approach is advantageous over conventional numerical techniques (e.g. trapezoidal rule) as it minimizes the effect of measurement error on calculated terms. LTD plasma concentration–time profiles were fitted to the following sum of exponential equation:

(9) $$C_{p}=\sum_{i=1}^{n}A_{i}\cdot e^{-\alpha_{i}\cdot t}$$

under the following constraints:

(10) $$A_{n}=-\sum_{i=1}^{n-1}A_{i}$$

(11) $$\alpha_{n}>\max\left(\alpha_{1},\alpha_{2},\ldots ,\alpha_{n-1}\right)$$

The number of exponential terms, *n*, was determined by non-linear regression analysis and comparison of Akaike Information Criterion (Akaike, 1974). The terms in Equation (8) were calculated as:

(12) $$\int_{0}^{t}C_{p}\mathrm{dt}=\sum_{i=1}^{n}A_{i}/\alpha_{i}\cdot \left(1-e^{-\alpha_{i}\cdot t}\right)$$

(13) $$\int_{0}^{\infty }C_{p}\mathrm{dt}=\sum_{i=1}^{n}A_{i}/\alpha_{i}$$

(14) $$k_{\mathrm{el}}=\min\left(\alpha_{1},\alpha_{2},\ldots ,\alpha_{n}\right)$$

The fraction of drug absorbed (F_abs_) obtained by deconvolution was correlated to the fraction of drug dissolved *in vitro* (F_diss_) using linear regression. Deconvolution using Wagner–Nelson method and IVIVC correlations were coded in *R* version 3.10 (R Core Team 2014, Vienna, Austria).

## Results and discussion

### Preliminary screening studies

Critical formulation and process variables in preparation of LTD transferosomes were screened using a low resolution Plackett–Burman design taking entrapment efficiency of LTD (EE) and PS of TRS (PS) as critical quality attributes (Supplementary Table S1). Analysis of the data collected according to the screening design (Table 1) identified surfactant type as the most critical variable influencing EE and PS (*p* < 0.005). The effect of spans on EE and PS was negative indicating that Span-60 was associated with higher EE and larger PS compared to Span-80. Our finding was in agreement with the finding of Yoshioka et al. (1994) who reported bigger niosomes with higher drug loading in case of Span-60. Although Span-60 and Span-80 share the same hydrophilic head structure and hydrocarbon chain length (C18), Span-80 contains unsaturated alkyl chain which makes it more permeable to drug leakage (De Gier et al., 1968) leading to lower entrapment ability for Span-80 (Hao et al., 2002; Mokhtar et al., 2008). In addition, Span-60 is solid at room temperature, as it has higher phase transition temperature (T_c_=53 °C) than Span-80 (T_c_= −12 °C). The higher phase transition temperature stabilizes the formed particles at room temperature, thus better maintains the entrapped drug (Yoshioka et al., 1994). As for vesicle size, the higher hydrophobicity of Span-80 (HLB = 4.3 versus HLB = 4.7 for Span-60) may explain its ability to produce smaller vesicles. Surface free energy at vesicular interface decreases as hydrophobicity of the surfactant increases (Wan & Lee, 1974). This phenomenon may also explain the significantly smaller particles obtained when the more hydrophobic tween, Tween-80 (HLB = 15 versus HLB = 16.4 for Tween-20), was used (Table 1). Using Tween-80 did not only decrease the size of the vesicles but also improved drug entrapment (Table 1).

**Table 1.** ANOVA of responses measured according to the Plackett–Burman design.

Factors	DF	SS	MS	*F*	*p* value	Effect
Entrapment efficiency						
Lipophilic surfactant	1	5434	5434	64.9	<0.001	−15.0
Ratio of lipid and edge activator to surfactant	1	430	430	5.13	0.036	−4.23
Sonication time	1	473	473	5.64	0.030	−4.44
Edge activator	1	91	91	1.09	0.312	−1.95
Ratio of lipid to edge activator	1	12	12	0.145	0.708	−0.71
Hydrophilic surfactant	1	1971	1971	23.5	<0.001	9.06
Residual	17	1424	84			
Particle size						
Lipophilic surfactant	1	135 300	135 300	16.3	0.001	−75.1
Ratio of lipid and edge activator to surfactant	1	470	470	0.057	0.814	−4.42
Sonication time	1	10 559	10 559	1.28	0.274	21.0
Edge activator	1	12 974	12 974	1.57	0.228	−23.3
Ratio of lipid to edge activator	1	117	117	0.014	0.907	−2.21
Hydrophilic surfactant	1	88 161	88 161	10.6	0.005	−60.6
Residual	17	140 813	8283			

DF: degrees of freedom; SS: sums of squared error; MS: mean squared error (MS = SS/DF); F: Fisher’s ratio (F = MS_Regression_/MS_Residual_).

Lipid and edge activator ratio to the lipophilic surfactant as well as sonication time exhibited moderate impact on EE (*p* < 0.05) and no impact on PS (*p* > 0.05). A negative influence for the ratio of lipid and edge activator to surfactant on EE was observed, suggesting that decreasing the ratio is better. Phospholipids can form micelle aggregates which lead to increased solubilization of drug and transferosomes (Cooper & Harirforoosh, 2014). Accordingly, increasing the ratio may produce leaky vesicles, thus decreases entrapment efficiency and increases PS.

Sonication time had disparate effects on the responses, negative on EE and positive on PS, suggesting that short times are associated with higher EE and smaller PS.

The quantity of the lipid and the type and quantity of the edge activator determines the transfersomal deformability and hence permeability (Ascenso et al., 2014). However, these variables proved to be useless predictors of transferosomal EE and PS (*p* > 0.05) (Table 1).

Based on the results of our preliminary study, we decided to: (i) use sodium cholate as the edge activator, (ii) fix the ratio of the lipid and edge activator to the hydrophobic surfactant at 2:1, (iii) fix the ratio of the lipid to the edge activator at 2:1, (iv) set sonication time to 10 min, (v) use Tween-80 as the hydrophilic surfactant, and (vi) optimize the composition of a ternary surfactant mixture consisting of Tween-80, Span-60, and Span-80.

### Optimization studies

The fact that surfactant type had the most significant influence on transferosomal EE and PS in the preliminary study drove us to optimize a ternary surfactant mixture consisting of Tween-80, Span-60, and Span-80. This need was further aggravated by the notice that the effects of the three surfactants on the response variables seemed to be conflicting (Table 1). Changing the composition of the ternary mixture resulted in EE range 9–63%, and PS range from 350 to more than 1000 nm (Supplementary Table S2). This substantial variability confirms the finding of the screening study that surfactants play a decisive role in transferosomal properties.

A simplex-centroid design constrained in such a way that all surfactants are represented in all blends (Figure 1, upper panel) was used to optimize the composition of the mixture. The design allowed efficient estimation of the effects of pure surfactants, as well as the effects of binary and ternary blends on PS and EE through MLR analysis. Composition–response relationships were best described by cubic and quadratic models for EE (*R^2^*=0.999) and PS (*R^2^*=0.997), respectively. Logarithmic transformation of the response variables further improved the model quality of fit where the *p* value of the overall *F*-statistic was reduced from 0.02 to 0.003 and from 0.01 to 0.0003 for EE and PS, respectively. The final models were:

(15) $$\log\mathrm{EE}=8\cdot X_{1}+4\cdot X_{2}+7\cdot X_{3}-26\cdot X_{1}X_{2}\;-26\cdot X_{1}X_{3}-23\cdot X_{2}X_{3}+161\cdot X_{1}X_{2}X_{3}$$

(16) $$\log\mathrm{PS}=6\cdot X_{1}+11\cdot X_{2}+6\cdot X_{3}-12\cdot X_{1}X_{2}\;+7\cdot X_{1}X_{3}-13\cdot X_{2}X_{3}$$

The final models shown in Equations (15) and (16) described the observed data adequately. ANOVA analysis of the final models (Supplementary Table S3) indicated that almost all terms were statistically significant (*p* < 0.05).

In the EE model (Equation (15)), the effects of pure blends were positive indicating that any of the three surfactants is associated with enhanced entrapment. Mixing the three components together produces a synergistic (i.e. leap forward) effect on the ability of the transferosomes to enclose more drug. This was evident by a significant positive coefficient of the tertiary blend term (Equation (15)). Spans alone produce highly hydrophobic vesicles characterized by rigid membrane that prevents drug leakage (Yoshioka et al., 1994). The large hydrophilic head of tween promotes loading more amount of the drug into the vesicles. Our result is in agreement with the findings of Gulati et al. (1998) who reported remarkable improvement in encapsulation efficiency of niosomes prepared using a mixture of Span-60 and Tween-60 rather than Span-60 alone.

Similar to EE, increasing the levels of the three surfactants increases the PS as evident by significant positive pure blend coefficients in Equation (16). Among the three surfactants, Span-60 was the most influential on PS (its effect was almost two-folds that of the other surfactants). However, addition of Span-80 or Tween-80 reverse the positive effect of Span-60 (i.e. produces leap backward effect), where binary blends involving Span-60 were associated with negative coefficients (Equation (16)). Solid lipid nanoparticles prepared using a mixture of surfactants is characterized by high stability and small PS when compared to those prepared using only one surfactant (Shah et al., 2015).

The ternary response surface plots shown in (Figure 1, lower panel) indicate that entrapment efficiency between 60 and 70% and PS < 400 nm are obtained as the ratio of the components approaches equality. Therefore, it was not surprising to see that the formulation associated with the highest desirability is similar to the central point formulation (M_7_). The optimal surfactant mixture is composed of 32% Tween-80, 33% Span-60, and 35% Span-80, indicating quasi-equal ratio mixture. When used in transferosome preparation, the associated EE, and PS were 60% and 380 nm, respectively. As predicted by the models depicted in Equations (15) and (16), the optimal formulation should exhibit EE of 62% and PS of 398 nm. Accordingly, the model prediction error (calculated as the percentage observed–predicted/observed) is 3.3 and 4.7% for EE, and PS, respectively. The extremely low prediction error values suggest a highly accurate model.

### Morphology of LTD-TRS

**Figure 2.**
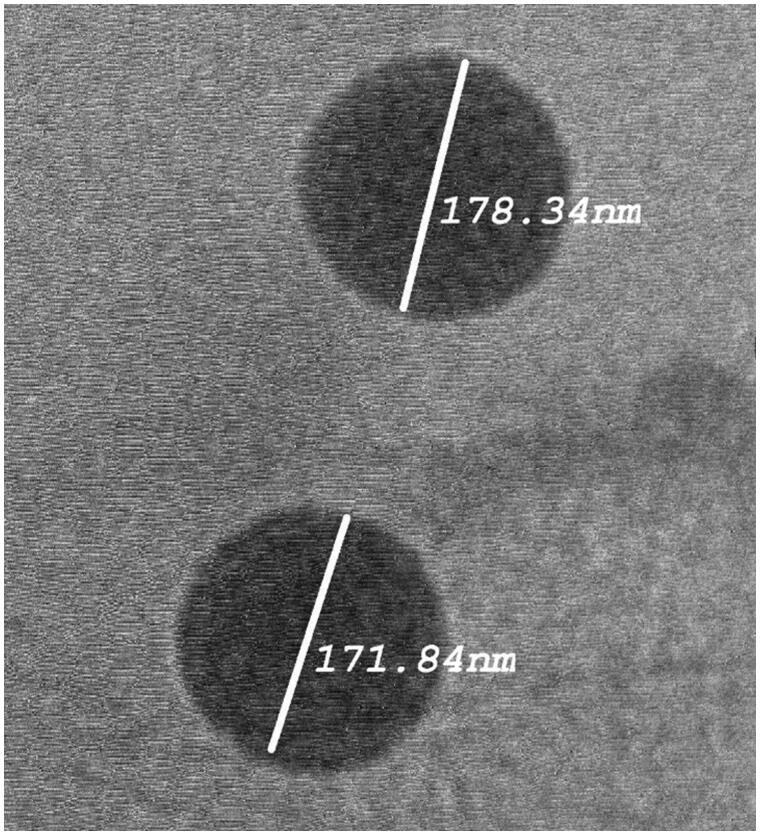
Transmission electron micrographs of optimized LTD-TRS formulation.

The morphology of the optimized formulation was observed using TEM. The TEM micrographs (Figure 2) showed vesicles with no aggregation and characterized by being spherical in shape with well identified outline and core. The vesicles showed smooth surface with narrow size distribution as revealed in Figure 2.

### Physical characterization of LTD-TRS-gel

Loading transferosomes into a gel base retards the movement and fusion of vesicles resulting in stable drug delivery system with better transbuccal delivery. The viscosity of prepared transfersomal gel was significantly larger than that of the control gel containing the untreated drug (14 755 and 10 356 cP, respectively). The higher viscosity of the LTD-TRS-gel is imparted by the lipid components of the transferosomal preparation (phosphatidyl choline, sodium cholate and spans) as previously claimed (Abdellatif & Tawfeek, 2015).

The pH value of any given formulation should be the same as the application site in order to avoid potential irritation (Ahad et al., 2016). Both the LTD-TRS gel and control gel showed pH of 6.8 resembling the pH of the buccal mucosa, thus precluding the possibility of intolerance. Both gels exhibited good homogeneity with no lumps.

### Muccoadhesion of LTD-TRS-gel

The weight required to detach both the transferosomal gel and control gel from the buccal mucosa was found to be 28 ± 0.2 g and 30 ± 0.5 g, respectively. The mucoadhesive strength for both formulations was close; 14 700 ± 2.5 and 13 720 ± 5.11 dyne/cm^2^, respectively. The slight increase in case of the LTD-TRS-gel may be attributed to the higher viscosity imparted by the transferosomal formulation.

### *In vitro* release studies

**Figure 3.**
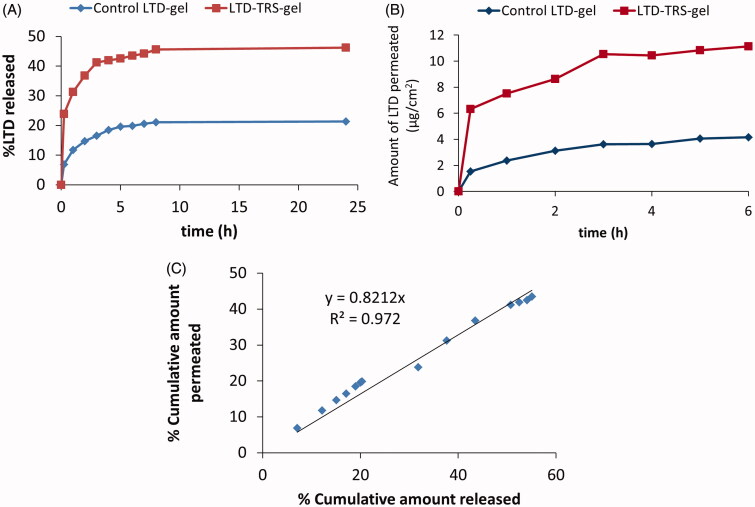
LTD *in vitro* release (A) and permeation across chicken buccal mucosa (B) from the transferosomal gel relative to the control gel. Plot (C) is correlation of percentage LTD permeated and released from the transferosomal and control gels.

LTD solubility decreases exponentially with pH increase (El-Hammadi & Awad, 2012). Therefore, LTD release in the oral cavity (at pH 6.8) may be a critical factor determining transbuccal permeability. LTD *in vitro* release profiles from the transferosomal and control gels are depicted in Figure 3(A). About 46 ± 3.5% of LTD was released from the transferosomal gels, compared to 24 ± 2.8% being released from the control gel within 24 h. The higher release rate from the LTD-TRS-gel is attributed to the submicron sized range of the transferosome vesicles and the existence of a blend of surfactant mixture, that allow partitioning of the drug from the vesicles as they get close to phospholipids bilayer. Enhancement of dissolution rate of water insoluble drugs by transferosome encapsulation has been reported in literature (Abdellatif & Tawfeek, 2015). Size of lipid-based vesicles plays a crucial role in controlling drug release rate. Elnaggar et al. (2011) found that seldinafil release from nanostructured lipid carriers (100 nm) was higher than that from solid lipid nanoparticles (180 nm).

The release profile of LTD from the transfersomal gel was biphasic where an initial fast release phase took place within the first 3 h followed by a sustained release phase that lasted for the rest of the study (Figure 3(A)). The biphasic release behavior is considered typical for drugs loaded into transferosomal vesicles (Ahmed, 2015). The rapid initial release phase results from the adsorbed free drug on the surface of the transferosomes.

LTD release data from the transferosomal gel was best fitted by the Higuchi diffusion model. This mean that release and permeation of LTD through membrane will be slow and last for several hours (Gupta et al., 2012).

### *Ex vivo* permeation studies

Cumulative amounts of LTD permeated across dissected chicken buccal mucosa against time profiles are shown in Figure 3(B). LTD flux from the transferosomal gel and control gel was 276 ± 16.74 and 92 ± 2.43 μg/cm^2^/h, respectively, achieving an enhancement ratio of 3. Several mechanisms explain the enhanced transbuccal permeation of LTD from the transferosomal preparation. Existence of a hydration gradient across the membrane layers constitutes a driving force on the hydrophilic phospholipids to move from low water location to the higher one present in the deeper layers of the membrane (Kulkarni, 2009). Due to presence of edge activators, the vesicles can deform themselves to penetrate through the very small pores of the membrane (Kulkarni, 2009). Moreover, the surfactant blend making up the transferosomes (spans and tweens) can loosen or fluidize the lipid bilayers of the membrane resulting in enhancement of vesicles permeability (Elnaggar et al., 2011). Finally, the small size of the vesicles increases the surface area of the formed film in contact to the membrane surface.

### *In vitro* release/*ex vivo* permeation correlation

Seeking determination of processes controlling LTD transbuccal permeability, the percent cumulative amount permeated *ex vivo* through chicken pouch was correlated to the percent cumulative amount diffused *in vitro* through dialysis membrane using regression (Figure 3(C)). The relationship for the transfesomal gel and control gel formulations pooled data was linear with correlation coefficient (*R^2^*) of 0.97. The slope of the regression lines was close to unity, indicating comparable increments in the percent dissolved and permeated. This result suggests that LTD buccal permeability may be limited by its solubility in the application site. Accordingly, the surge in LTD flux from the LTD-TRS-gel (Figure 3(B)) is not solely explained by the ability of the transferosomes to squeeze themselves into deeper layers of biological membranes as generally accepted (Kulkarni, 2009), but also, at least in part, by improving solubility of the poorly soluble drug in the surrounding biological fluid (Figure 3(A)).

### *In vivo* pharmacokinetic study in healthy human volunteers

**Figure 4.**
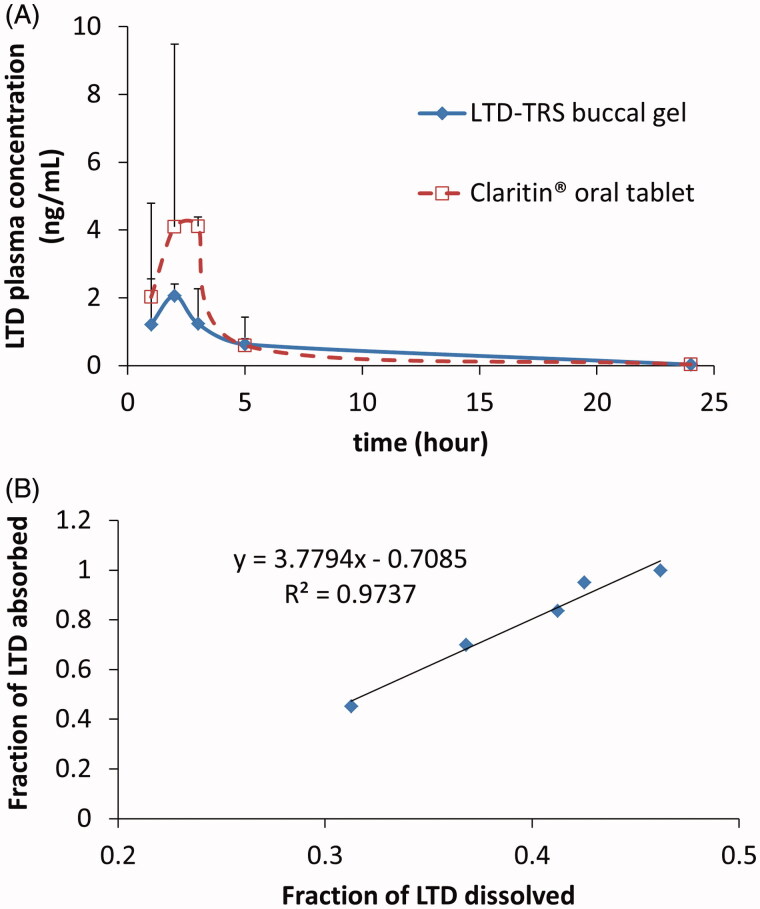
Average (+SD) Plasma LTD cementations following administration of 10 mg LTD in transferosomal buccal gel and marketed Claritin® tablet in three healthy human volunteers (A), and Level A IVIVC plot for LTD in the transferosomal buccal gel (B). Plasma concentrations of both formulations at each time point in plot (A) were not significantly different on ANOVA test (*p* > 0.05).

Figure 4(A) depicts the average plasma concentration versus time profiles of LTD obtained after single administration of both the transferosomal buccal gel and the marketed oral tablet. The estimates of the mean pharmacokinetic parameters obtained by non-compartmental fitting of the concentration–time data of LTD with the associated variability parameters are illustrated in Table 2. Claritin® tablets exhibit larger *C*_max_ and AUC values. However, statistical analysis of the parameters of both formulations failed to detect significant difference. The lack of significance is attributed to the marked variability in the measured concentrations of Claritin® when administered orally (Figure 4(A)). The failure to capture significance reflects similar bioavailability or may be an artifact associated with the small sample size used in the bioavailability study.

**Table 2.** Pharmacokinetic parameters of LTD after buccal administration of the transferosomal gel and the marketed tablet (Claritin®) in 10 mg doses to three healthy human volunteers.

Parameter	LTD-TRS buccal gel	Claritin® oral tablets	ANOVA *p* value
*C*_max_ (ng/mL)	2.13 ± 0.25 (11.5)	5.90 ± 2.83 (48)	>0.05
*T*_max_ (h)	2.50 ± 0.71 (28.3)	2.50 ± 0.71 (28.3)	>0.05
AUC_0–24_ (ng h/mL)	12.1 ± 1.11 (9.22)	18.8 ± 17.9 (94.8)	>0.05
AUC_0–∞_ (ng h/mL)	12.4 ± 0.679 (5.48)	19.1 ± 18.1 (95.3)	>0.05
MRT (h)	4.40 ± 0.52 (11.8)	3.34 ± 0.577 (17.3)	>0.05
*t*_0.5_ (h)	3.94 ± 3.86 (97.8)	2.77 ± 1.59 (57.4)	>0.05

Values are mean ± SD (%CV). %CV is percentage coefficient of variation (calculated as SD/mean × 100).

Using the buccal route reduced the %CV of *C*_max_ and AUC by 76, and 90%, respectively. LTD is mainly metabolized to its major metabolite, descarboethoxyloratadine, by CYP3A4 and CYP2D6 enzymes (Yumibe et al., 1996) which are expressed in human buccal tissue. However, it has been suggested that its expression and catalytic activity in buccal epithelium is limited (Vondracek et al., 2001). This fact may explain the diminished between-subject variability in the case of LTD-TRS-gel.

### IVIVC

Recommended by regulatory authorities, IVIVC is a valuable tool for predicting *in vivo* results based on *in vitro* data and can be used as a surrogate for further bioequivalence studies (Uppoor, 2001). The point-to-point correlation of percentage LTD release from the transferosomal gel to fraction absorbed *in vivo* was excellent as evidenced by correlation coefficient >0.97 (Figure 4(B)).

## Conclusion

A transferosomal gel formulation for buccal administration was successfully developed for LTD. The composition of hydrophilic–hydrophobic surfactant mixture plays a decisive role in controlling transferosome PS and EE. Optimal transferosomes characterized by submicron size (380 nm), spherical shape and adequate loading capacity (60%) were obtained by using quasi-equal ratio surfactant mixture. In terms of amount permeated, percentage released, and mucoadhesion time, the transferosomal gel proved superior to control, transferosome-free gel. Bioavailability of the transferosomal gel was comparable to Claritin® oral tablets. However, inter-individual variability in absorption parameters was considerably reduced when the buccal gel was used. In summary, a novel buccal delivery system for LTD was developed. However, further clinical investigation is warranted to evaluate its therapeutic effectiveness and utility.

## Supplementary Material

IDRD_Mohammed_et_al_Supplemental_Content.docx

## Notes

**Supplementary material available online**
