# Supplementary material for: Loratadine bioavailability via buccal transferosomal gel: formulation, statistical optimization, in vitro/in vivo characterization, and pharmacokinetics in human volunteers
Source: Drug Deliv. 2017 May 8;24(1):781–91. doi: 10.1080/10717544.2017.1321061 (PMC8241167; doi:10.1080/10717544.2017.1321061)
Supplement: IDRD_Mohammed_et_al_Supplemental_Content.docx [file IDRD_A_1321061_SM7539.docx]

**Supplementary materials**

**Table S1:** Entrapment efficiency and particle size of Loratadine transferosome trial formulations suggested by the Plackett-Burman design. Factor coding is shown in parentheses.

| **Formulation** | **Factors** | | | | | | **Response** | |
| --- | --- | --- | --- | --- | --- | --- | --- | --- |
|  | Lipophilic surfactant | Ratio of lipid and edge activator to surfactant | Sonication time (min) | Edge activator | Ratio of lipid to edge activator | Hydrophilic surfactant | Entrapment efficiency (% ) | Particle size (nm) |
| T_1_ | span 60 (-1) | 2:1 (-1) | 10 (-1) | sodium cholate (-1) | 2:1 (-1) | tween 20 (-1) | 51.2±0.29 | 615.5±0.42 |
| T_2_ | span 80 (+1) | 4:1 (+1) | 10 (-1) | sodium deoxy cholate (+1) | 2:1 (-1) | tween 20 (-1) | 1.39±0.59 | 542.9±0.49 |
| T_3_ | span 60 (-1) | 2:1 (-1) | 10 (-1) | sodium deoxy cholate (+1) | 5:1 (+1) | tween 80 (+1) | 60.7±0.42 | 501.8±0.57 |
| T_4_ | span 80 (+1) | 2:1 (-1) | 10 (-1) | sodium cholate (-1) | 5:1 (+1) | tween 80 (+1) | 24.6±0.50 | 370.2±0.28 |
| T_5_ | span 80 (+1) | 4:1 (+1) | 10 (-1) | sodium deoxy cholate (+1) | 5:1 (+1) | tween 20 (-1) | 0.10±0.14 | 401.5±0.28 |
| T_6_ | span 60 (-1) | 4:1 (+1) | 30 (+1) | sodium cholate (-1) | 5:1 (+1) | tween 20 (-1) | 11.2±0.39 | 606.5±0.64 |
| T_7_ | span 80 (+1) | 2:1 (-1) | 30 (+1) | sodium deoxy cholate (+1) | 2:1 (-1) | tween 80 (+1) | 7.15±0.21 | 341.7±0.28 |
| T_8_ | span 60 (-1) | 4:1 (+1) | 30 (+1) | sodium deoxy cholate (+1) | 2:1 (-1) | tween 80 (+1) | 46.6±0.43 | 473.1±0.14 |
| T_9_ | span 80 (+1) | 2:1 (-1) | 30 (+1) | sodium cholate (-1) | 2:1 (-1) | tween 20 (-1) | 9.32±0.45 | 597.8±0.85 |
| T_10_ | span 80 (+1) | 4:1 (+1) | 30 (+1) | sodium cholate (-1) | 5:1 (+1) | tween 80 (+1) | 26.7±0.31 | 507.3±0.35 |
| T_11_ | span 60 (-1) | 4:1 (+1) | 10 (-1) | sodium cholate (-1) | 2:1 (-1) | tween 80 (+1) | 48.2±0.23 | 654.1±1.41 |
| T_12_ | span 60 (-1) | 2:1 (-1) | 30 (+1) | sodium deoxy cholate (+1) | 5:1 (+1) | tween 20 (-1) | 32.0±0.35 | 811.4±0.92 |

*Results are mean±SD

**Table S2**: Amounts of the surfactants in a 1.2 gram ternary mixture according to the constrained simplex-centroid design with the corresponding transferosome formulation lipid and edge activator amounts, entrapment efficiency (EE) and particle size (PS).

| **Mixture** | **Surfactant amount (g)** | | | **Lipid and edge activator amounts (g)** | | **Response** | |
| --- | --- | --- | --- | --- | --- | --- | --- |
|  | **X_1_: tween 80** | **X_2_: span 60** | **X_3_: span 80** | **phosphatidyl choline** | **sodium cholate** | **EE (%)** | **PS (nm)** |
| M_1_ | 0.5 | 0.2 | 0.5 | 1.4 | 0.7 | 41.8±0.14 | 801±0.35 |
| M_2_ | 0.8 | 0.2 | 0.2 | 0.8 | 0.4 | 43.4±0.54 | 508±0.35 |
| M_3_ | 0.2 | 0.5 | 0.5 | 2.0 | 1.0 | 24.3±0.3 | 357±0.35 |
| M_4_ | 0.2 | 0.2 | 0.8 | 2.0 | 1.0 | 36.1±0.22 | 465±0.35 |
| M_5_ | 0.5 | 0.5 | 0.2 | 1.4 | 0.7 | 22.1±0.37 | 391±0.49 |
| M_6_ | 0.2 | 0.8 | 0.2 | 2.0 | 1.0 | 9.76±0.64 | 1124±0.21 |
| M_7_ (1) | 0.4 | 0.4 | 0.4 | 1.6 | 0.8 | 62.7±0.60 | 351±0.50 |
| M_7_ (2) | 0.4 | 0.4 | 0.4 | 1.6 | 0.8 | 61.3±0.09 | 392±0.35 |

*Response values are mean±SD.

*Ratio of lipid and edge activator to hydrophobic surfactant was fixed to 2:1.

*Ratio of lipid to edge activator was fixed to 2:1.

*Sonication was performed for 10 minutes.

**Table S3:** Analysis of variance of the final models for measured responses.

| **Parameters** | **DF** | **SS** | **MS** | **F** | ***p*-value** |
| --- | --- | --- | --- | --- | --- |
| **Entrapment efficiency (EE)** |  |  |  |  |  |
| X_1_ | 1 | 84.3 | 84.3 | 333259 | 0.001 |
| X_2_ | 1 | 6.4 | 6.4 | 25456 | 0.004 |
| X_3_ | 1 | 8.1 | 8.1 | 32063 | 0.004 |
| X_1_X_2_ | 1 | 0.2 | 0.2 | 680 | 0.024 |
| X_1_X_3_ | 1 | 0.2 | 0.2 | 601 | 0.026 |
| X_2_X_3_ | 1 | 0.3 | 0.3 | 1255 | 0.018 |
| X_1_X_2_X_3_ | 1 | 0.5 | 0.5 | 1938 | 0.014 |
| Regression | 7 | 100.0 | 14.3 | 56465 | 0.003 |
| Residual | 1 | 0.0003 | 0.0003 |  |  |
| Total | 8 | 100.0 | 12.5 |  |  |
| **Particle size (PS)** |  |  |  |  |  |
| X_1_ | 1 | 249 | 249 | 16369 | <0.0001 |
| X_2_ | 1 | 45 | 45 | 2947 | <0.0001 |
| X_3_ | 1 | 15 | 15 | 1000 | 0.001 |
| X_1_X_2_ | 1 | 0.46 | 0.46 | 30 | 0.032 |
| X_1_X_3_ | 1 | 0.13 | 0.13 | 9 | 0.098 |
| X_2_X_3_ | 1 | 0.50 | 0.50 | 33 | 0.029 |
| Regression | 6 | 311 | 52 | 3398 | <0.0001 |
| Residual | 2 | 0.03 | 0.02 |  |  |
| Total | 8 | 311 | 39 |  |  |

DF: degrees of freedom; SS: sums of squared error; MS: mean squared error (MS = SS/DF); F: Fisher’s ratio (F = MS_Regression_/MS_Residual_), X_1_: tween 80; X_2_: span 60; X_3_: span 80.
